# A study of high dose furmonertinib in EGFR exon 20 insertion mutation-positive advanced non-small cell lung cancer

**DOI:** 10.3389/fonc.2024.1314301

**Published:** 2024-04-08

**Authors:** Song Hu, Hao Ming, Qian He, Ming Ding, Hao Ding, Chong Li

**Affiliations:** ^1^ Department of Respiratory and Critical Care Medicine, Third Affiliated Hospital of Soochow University, Changzhou, China; ^2^ Department of Respiratory and Critical Care Medicine, Affiliated Hospital of Jiangsu University, Zhenjiang, China; ^3^ Division of Respiratory Disease, Affiliated People’s Hospital of Jiangsu University, Zhenjiang, China

**Keywords:** the epidermal growth factor receptor, exon 20 insertion, furmonertinib, non-small cell lung cancer, first-line treatment

## Abstract

**Background:**

The epidermal growth factor receptor (EGFR) ex20ins mutation, as a rare subtype of mutation, has gradually attracted attention. Its heterogeneity is high, its prognosis is extremely poor, and the efficacy of existing traditional treatment plans is limited. In this study, we aimed to evaluate efficacy of high dose furmonertinib as a first-line treatment for EGFR ex20ins-positive NSCLC.

**Methods:**

This is a retrospective, multi-center, non-interventional study. From May 2021 to March 2023, 9 NSCLC patients with EGFR ex20ins were enrolled. Efficacy and safety of 160 mg furmonertinib were evaluated. Objective response rate (ORR), disease control rate (DCR), median progression-free survival (PFS) and treatment related adverse events (TRAEs) were assessed.

**Results:**

Of the evaluated patients, six patients experienced partial remission (PR), two patients experienced stable disease (SD) and one patient experienced progress disease (PD). Data indicated 66.7% ORR and 88.9% DCR. The median progression free survival (PFS) was 7.2 months (95% CI: 6.616 - 7.784). Besides, a longgest PFS with 18 months was found in one patient with p.H773_V774insGTNPH mutation. No ≥ level 3 adverse events have been found.

**Conclusions:**

The study proved the potential efficacy of 160mg furmonertinib in patients with advanced NSCLC with EGFR ex20ins. Meanwhile, 160mg furmonertinib had a good safety profile.

## 1 Introduction

According to the global cancer statistics in 2020, the incidence of lung cancer ranks second in the world, with 2.2 million new cases per year, accounting for 11.4% of the total number of new tumors, and the death rate of lung cancer is the first cause of cancer death, accounting for 14.3% of the total number of tumor deaths (1). Lung cancer has become one of the most tumors threatening human health, among which non-small cell lung cancer (NSCLC) is the main pathological type of lung cancer, accounting for 80%-85%, and most patients are found to be in advanced stages of the disease. Platinum-based chemotherapy is the main treatment for advanced NSCLC. In recent years, the epidermal growth factor receptor (EGFR) is gradually being recognized. Multiple studies have shown that targeted therapy, represented by EGFR tyrosine kinase inhibitors (EGFR-TKIs), not only significantly prolongs the progression free survival (PFS) of patients with advanced NSCLC, but also improves their quality of life, which has revolutionized the treatment of patients with advanced NSCLC.

The EGFR gene is cmposed of 28 exons, and most of the mutations occur in exons 18-21. Common mutations include exon 19 inframe deletion (ex19del) and exon 21 L858R alterations (ex21L858R), which accounts for 80%-85% of all EGFR mutations (2). These two classical EGFR mutations have good sensitivity to first-generation EGFR-TKIs (erlotinib, gefitinib), second-generation EGFR-TKIs (afatinib, dacotinib) and third-generation EGFR-TKIs (osimertinib) drugs. The remaining EGFR mutations are known as rare mutations. Among rare mutations, EGFR ex20ins mutation is the most common. The incidence of EGFR ex20ins in EGFR mutated NSCLC patients is 4% -12%, while in NSCLC patients, the incidence is 1.8% -2.3% (3).

Before Mobosetinib and Amivantamab were approved for EGFR ex20ins NSCLC by FDA, the main treatment for patients with EGFR ex20ins mutation were traditional EGFR-TKIs, platinum-containing chemotherapy and immunotherapy. However, these treatment options have limited benefits (4–6). Hence, better treatment plans are needed for these patients.

Furmonertinib is an irreversible, selective, third-generation EGFR-TKI. The FURLONG study (NCT03787992) (7) showed that first-line treatment with furmonertinib in Chinese patients with advanced EGFR gene mutation NSCLC resulted in a median PFS of 20.8 months, which was 9.7 months longer than the 11.1 months in the gefitinib group. In terms of safety, the median drug exposure time of patients in the furmonertinib group was longer than that in the gifitinib group (18.3 months vs. 11.2 months), but the incidence of ≥ Level 3 adverse events in the furmonertinib group was lower than that in the gifitinib group (11% vs. 18%). On March 3, 2021, the National Medical Products Administration (NMPA) approved the use of furmonertinibb for second-line treatment in adult patients with locally advanced or metastatic NSCLC carrying EGFR 20 exon T790M (T790M) resistance mutations. Nowadays, the efficacy of furmonertinib as a first-line treatment for EGFR ex20ins-positive NSCLC is currently being studied. Preclinical data and Phase Ib study results published by furmonertinib showed that receiving 240 mg/d of furmonertinib treatment resulted in an ORR of 60% and a DCR of 100%, with no ≥ grade 3 adverse events occurring (8). However, there is still a lack of results about the effect of 160 mg/d of furmonertinib on EGFR ex20ins-positive NSCLC.

In this study, we retrospectively analyzed the efficacy and safety of first-line treatment with 160 mg/d of furmonertinib in patients with advanced NSCLC harboring EGFR ex20ins mutations. We also collected and analyzed the clinical pathological and molecular characteristics of NSCLC patients with EGFR ex20ins mutations.

## 2 Material and methods

### 2.1 Study design

This was a retrospective, multi-center, non-interventional study. Patients received furmonertinib 160mg once daily for first-line treatment. We collected the clinical information of patients, including age, sex, pathological type, smoking history, metastatic site, gene status, efficacy assessment, and adverse events (AEs). Our study was approved by the ethics committee of the First Hospital of Changzhou.

### 2.2 Inclusion criteria

a) Non-small cell lung cancer (NSCLC) diagnosed by histology or cytology at the age of 18 or older. b) Tumor driven gene testing has confirmed the presence of EGFR20 exon insertion mutations in tissue or blood samples. c) Stage IV NSCLC patients who did not undergo any systemic treatment before their first medication. d) Having at least one measurable tumor lesion (according to RECIST 1.1). e) The ECOG score was 0-1, and there was no significant deterioration of the disease within the two weeks prior to screening.

### 2.3 Efficacy and safety assessments

All patients who received at least one dose of furmonertinib were included in the efficacy and safety assessment. Patients with measurable disease at baseline and had been re-examined were evaluated for efficacy analysis. Radiographic tumor assessments were completed every 4–8 weeks. Response was assessed by the investigators according to the Response Evaluation Criteria in Solid Tumors (RECIST), version 1.1. Adverse events were recorded from the clinical data. The PFS was calculated from the first day of treatment with furmonertinib to disease progression or death or the last follow-up visit. Adverse events were graded according to the NCI Common Terminology Criteria for Adverse Events (CTCAE), version 4.0.

### 2.4 Statistical analyses

The descriptive statistics for clinical and demographic characteristics were summarized with numbers and percentages of categorical variables. The ORR and DCR were indicated by the rate of responses. The PFS were calculated by Kaplan-Meier method. All statistical analyses were performed using SPSS software version 26.0 (IBM Corp., NY, USA).

## 3 Results

### 3.1 Patient characteristics

Between May 2021 to March 2023, 9 patients were enrolled in this study. The characteristics of 9 patients evaluated in this study are summarized in **Table 1**. Median age was 59 years (range 49–75), 33.3% (n=3) of patients were female, 66.7% (n=6) of patients were male, and 44.4% (n=4) were never-smokers. The most common sites of metastasis at baseline were bone (55.6%) and lung (44.4%). In addition, the most common progressive lesion is brain metastasis.

**Table 1:** Basic characteristics of the patients included in this study.

	No. of patients	(%)
Total enrolled	9	
Aged (years)		
Median(range)	59(49-75)	
Sex		
Male	6	66.7
Female	3	33.3
ECOG performance status (pre-treatment)		
0	6	66.7
1	3	33.3
Histology		
Adenocarcinoma	8	88.9
Squamous	1	11.1
Disease stage		
IV A	1	11.1
IV B	8	88.9
Site of metastasis		
Bone	5	55.6
Pleura	3	33.3
Lung	4	44.4
adrenal	1	11.2
Central nervous system	2	22.2
smoking history		
Yes	5	55.6
No	4	44.4
EGFR mutation status		
p.H773_V774insGTNPH	1	11.1
p.P772_H773insT	1	11.1
p.S768_D770dup	3	33.3
p.P772_H773insPNP	1	11.1
p.V769_D770insCV	1	11.1
p.P771_P772insT	2	22.2

ECOG, Eastern Cooperative Oncology Group.

### 3.2 Molecular characterization of EGFR ex20ins mutations

**Figure 1:**
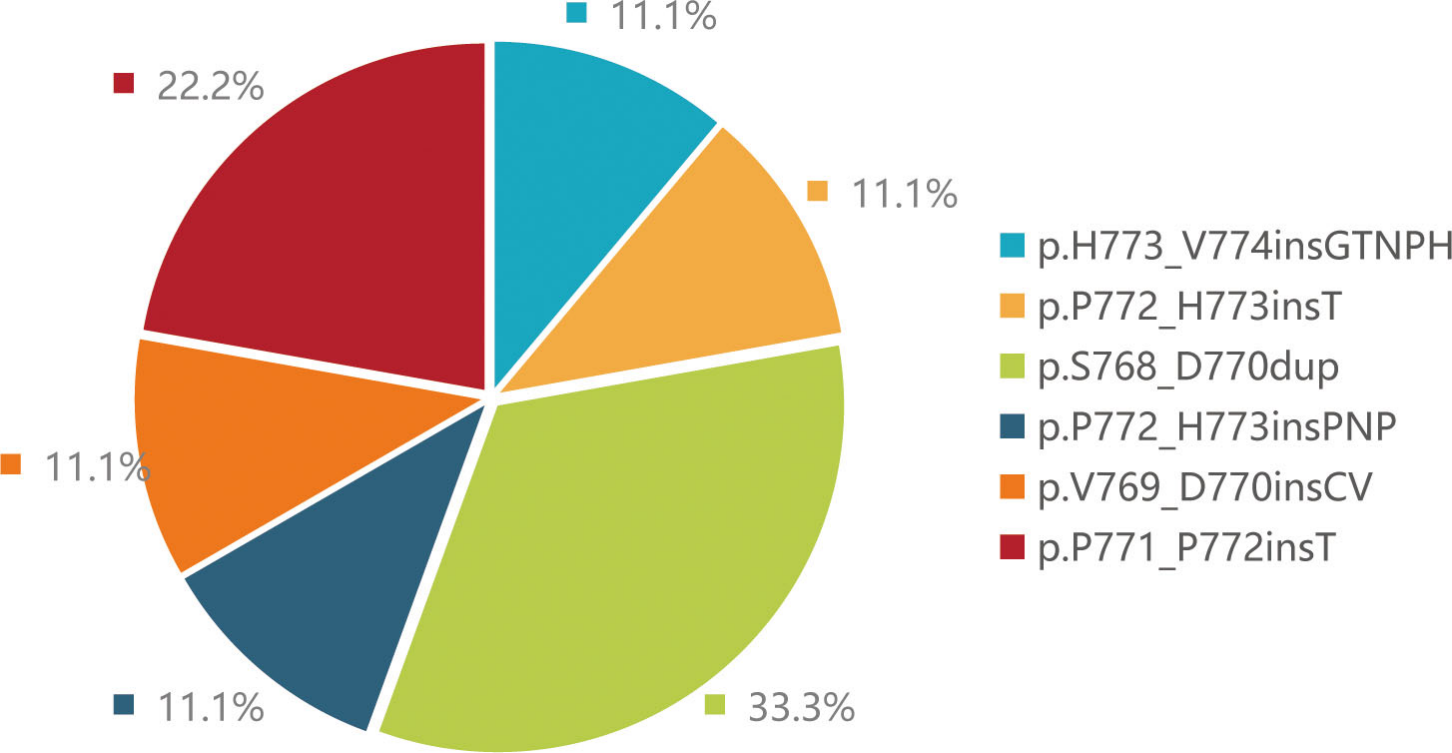
Proportion of EGFR ex20ins mutation subtypes identified by next-generation sequencing.

**Figure 2:**
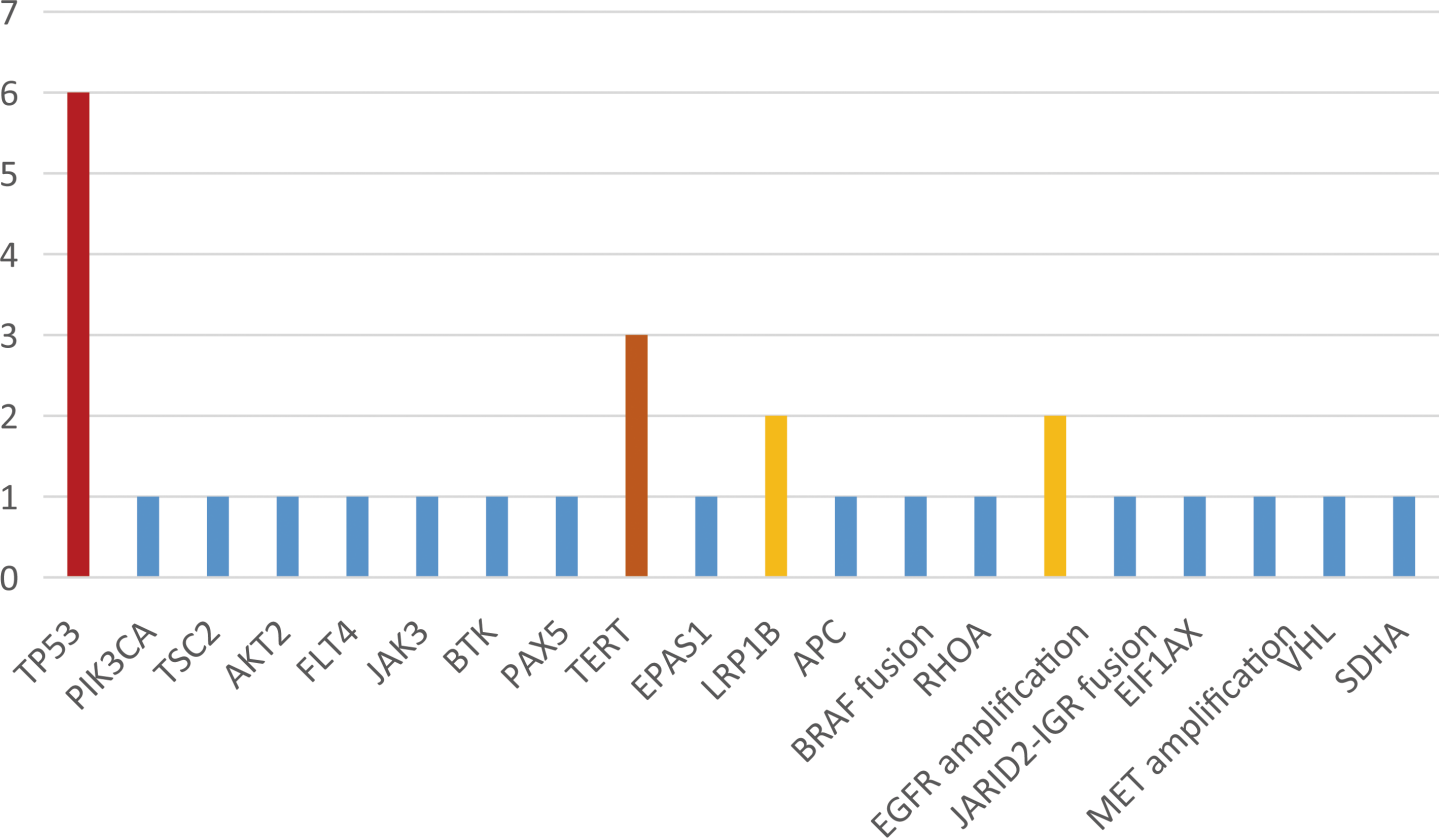
Distribution of co-mutation identified by next-generation sequencing.

NGS (Next-generation sequencing technology) analysis determined the mutation types of 9 patients. Three patients harbored EGFR p.S768_D770dup, two patients harbored p.P771_P772insT, one patient harbored p.H773_V774insGTNPH, one patient harbored p.P772_H773insT, one patient harbored p.V769_D770insCV and one patient harbored p.P772_H773insPNP mutations (**Figure 1**). Besides, more than 20 co-mutations detected by NGS and the most common co-mutation is TP53 (66.7%), TERT (33.3%) (**Figure 2**).

### 3.3 Efficacy analysis

**Figure 3:**
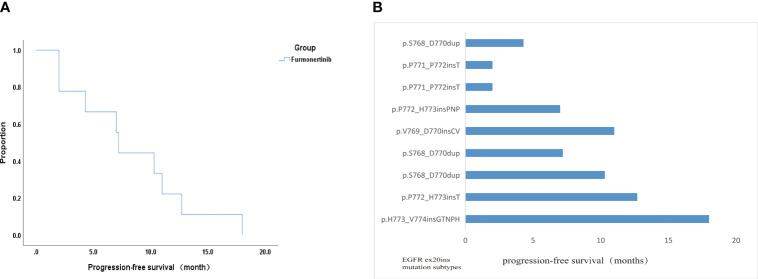
Efficacy analysis data of this study. **(A)** Kaplan-Meier curve displaying overall survival. **(B)** PFS of all patients with the EGFR ex20ins mutations in this study.

Of the nine evaluated patients, six patients experienced PR, two patients experienced SD and one patient experienced PD. These data indicated 66.7% ORR and 88.9% DCR. Besides, median PFS time was 7.2 months (95% CI: 6.616 - 7.784) (**Figure 3A**). PFS of one patients with H773_V774insGTNPH is up to 18 months. In four patients with H773_V774insGTNPH, S768_D770dup, V769_D770insCV and P772_H773insT, lung cancer remained stable for more than 10 months. In two patients with p.P771_P772insT, the tumor was progressing rapidly. Besides, in two patients without co-mutation, they each obtained longer PFS(18 months and 12.7 months) (**Figure 3B**). These data indicate variable efficacy pattern among patients with EGFR ex20ins positive NSCLC.

### 3.4 Safety analysis

In general, furmonertinib treatment was well tolerated. The safety profile of this study is summarized in **Table 2**. Increased γ-glutamyl transpeptidase (GGT) was observed in one patient. Patient’s liver function returns to normal after dose reduction to 120 mg once daily. One patient developed diarrhea and one patient developedoral ulcers. In summary, safety profile of furmonertinib 160 mg is well accepted.

**Table 2:** Treatment-related adverse events.

TRAEs,n (%)	Grades 1–2	Grades 3–5
Diarrhea	1	0
Oral ulcer	1	0
Liver function damage	1	0

## 4 Discussion

The majority of EGFR ex20ins mutations occur on the C-terminal loop after the C-helix, with a few occurring within the C-helix (9). According to the different positions of insertion mutations, EGFR ex20ins mutations can be divided into C-helix insertion, proximal insertion, and distal insertion. In our study, mutations in eights patients were located in near-loop and just one patients was located in far-loop. It is worth noting that EGFR ex20ins has strong molecular heterogeneity. To date, over 100 EGFR ex20ins variant types have been reported (10, 11). Studies shown that the most frequent variant was A767_V769dup (34.9%), S768_D770dup (15.5%) and N771_H773dup (6.2%). However, our study shown that the most frequent variant was S768_D770dup (33.3%) and P771_P772insT (22.2%), which may be related with a smaller sample size of our study. We also found that the most frequent co-mutation were TP53 (66.7%), which was consistent with literature (12, 13). As we know, TP53 is closely related to prognosis of advanced NSCLC with EGFR mutation. A study showed that TP53 was associated with faster resistance in EGFR-mutant NSCLC and mediated acquisition of resistance mutations to EGFR tyrosine kinase inhibitors (14). In our study, we also found that neither of the two patients with the longest PFS had TP53 mutations. However more cases are needed to ensure the impact of co-occurring TP53 mutations.

In the real-world, the first-line treatment of patients with advanced EGFR ex20ins NSCLC mostly use chemotherapy. A retrospective study showed that the ORR and DCR at 6 months of advanced NSCLC patients with EGFR ex20ins treated with first-line platinum based chemotherapy were 19.2% and 41.3% (10). The median PFS was 6.4 months. The first generation TKI drugs (gefitinib, erlotinib) are a reversible ATP competitive inhibitor that can selectively inhibit the ex19del and ex21L858R mutant EGFR, but have almost no effect on the ex20ins mutation. Previous studies have shown that the median PFS of advanced NSCLC patients with EGFR ex20ins mutations receiving first-generation EGFR-TKIs treatment was only 1.9 months (15). As the third-generation orally highly efficient and irreversible EGFR-TKIs, the therapeutic effect of osimitinib on EGFR ex20ins is quite controversial in various studies. A small sample study reported that six advanced EGFR ex20ins NSCLC patients treated with osimitinib with an objective response rate (ORR) of 100%, mPFS of 6. 2 months and a good safety profile (16). A single arm phase II study was conducted on NSCLC patients with EGFR ex20ins mutation, in which the dose of osimitinib was increased to 160 mg. The results showed that the ORR of 20 patients was 25%, with a median PFS of 9.7 months and a median DOR was 5.7 months (17). This result indicates that high-dose osetinib can be used as a treatment option for patients with good tolerance. However, there are unreasonable aspects to this research data, such as the median DOR is much smaller than the median PFS. Considering that the survival curve of small sample studies often presents a stepped shape, it indicates that the estimation of median PFS may not be stable. Besides, VAN VEGGEL et al. (18) found that osimitinib had limited effect on EGFR ex20ins NSCLC. After increasing the dose of osimitinib, they found that the ORR was 6.5% and median PFS was only 2.3 months, which did not show a better treatment effect. In September 2021, the FDA accelerated the approval of mobocertinib for the treatment of locally advanced or metastatic NSCLC patients with EGFR ex20ins mutations confirmed by FDA approved testing methods during or after platinum chemotherapy. The data from a phase I/II multicenter study (NCT02716116) showed that among advanced EGFR ex20ins mutant NSCLC patients who had previously received containing platinum chemotherapy, the ORR receiving mobocertinib (160 mg/d) was 43%, the median DOR was 13.9 months, the DCR was 86%, and the median PFS was 7.3 months (19). Another Phase I/II study published at the 2022 European Society for Medical Oncology (ESMO) conference showed a confirmed ORR of 28.1%, DCR of 78%, median PFS of 7.3 months, and median OS of 20.2 months (20). However, it lacks more data on first-line treatment.

Furmonertinib is one of the third generation EGFR-TKIs drug. The preclinical studies showed that the half-maximal inhibitory concentration (IC50) of furmonertinib for EGFR ex20ins type was 5-10 times lower than that of EGFR wild, which indicates furmonertinib has shown encouraging anti-tumor activity in EGFR 20ins (8). The FAVOUR Ib phase study targeting EGFR ex20ins mutant patients showed that the ORR of first-line treatment with furmonertinib 240 mg was 69%, the DCR was 96.6%, and the median PFS was 10.7 months (21). Another study targeting EGFR ex20ins mutant NSCLC patients with ≥ 2 lines showed that the overall ORR and DCR of 15 patients receiving furmonertinib treatment were 53.5%, 100%, and the 3-month PFS rate was 100% (22). Moreover, both of the above studies have shown good safety and tolerability of furmonertinib, and no ≥ level 3 adverse events have been found. However, they did not explore the efficacy of the furmonertinib 160mg as a first-line treatment on EGFR ex20ins mutant patients. In our study, we found that the ORR of first-line treatment with furmonertinib 160 mg was 66.7%, the DCR was 88.9% and the median PFS was 7.2 months (95% CI: 6.616 - 7.784)months. Our results showed the first-line treatment efficacy of furmonertinib was superior to traditional chemotherapy and classic EGFR-TKI drugs, such as gefitinib, erlotinib and osimitinib. Moreover, compared with furmonertinib 240 mg daily, our study showed that furmonertinib 160 mg daily also could produce good therapeutic effects on EGFR ex20ins mutant patients.

As we known, EGFR ex20ins has strong molecular heterogeneity and different insertion sites result in different drug efficacy. John V Heymach et al. found that poziotinib sensitivity was highly dependent on the insertion location, with near-loop insertions (amino acids A767 to P772) being more sensitive than far-loop insertions (23). In our study, we also have an interesting discovery that the PFS of one patient with p.H773_V774insGTNPH is up to 18 months. We noticed that the patient only had an EGFR ex20ins mutation and there is no concomitant mutation, which may be one of the reasons why patients have long PFS. Moreover, we noticed that the patients with N771_H772insT are more likely to develop central nervous system metastases and have poor response to furmonertinib. So far, no literature has reported this result. This result also requires a larger sample size to confirm. Hence, we can pay more attentions on the therapeutic effect of furmonertinib on different insertion location of EGFR ex20ins.

In summary, this study has demonstrated that furmonertinib had good anti-tumor activity and tolerance in NSCLC patients with EGFR ex20ins mutation. In first-line treatment, it is superior to traditional targeted drugs and platinum containing chemotherapy regimens. Therefore, furmonertinib may be as a first-line treatment option for patients with advanced EGFR ex20ins NSCLC. In addition, the efficacy of furmonertinib may associated with different EGFR ex20ins variant types.

## 5 Conclusions

The study proved the potential efficacy of 160mg furmonertinib in patients with advanced NSCLC with EGFR ex20ins. Meanwhile, 160mg furmonertinib had a good safety profile.

## Data availability statement

The original contributions presented in the study are included in the article/supplementary material. Further inquiries can be directed to the corresponding authors.

## Ethics statement

The studies involving humans were approved by the ethics committee of the First Hospital of Changzhou. The studies were conducted in accordance with the local legislation and institutional requirements. Written informed consent for participation was not required from the participants or the participants’ legal guardians/next of kin because this is a retrospective, multi-center, non-interventional study.

## Author contributions

SH: Data curation, Writing – original draft. HM: Data curation, Writing – original draft. QH: Writing – original draft. MD: Writing – review & editing, Data curation. HD: Methodology, Writing – review & editing. CL: Methodology, Writing – review & editing.

## References

[1] Sung H Ferlay J Siegel RL Laversanne M Soerjomataram I Jemal A. Global cancer statistics 2020: GLOBOCAN estimates of incidence and mortality worldwide for 36 cancers in 185 countries. CA Cancer J Clin. (2021) 71:209–49. doi: 10.3322/caac.21660 33538338

[2] Batra U Biswas B Prabhash K Krishna MV. Differential clinicopathological features, treatments and outcomes in patients with Exon 19 deletion and Exon 21 L858R EGFR mutation-positive adenocarcinoma non-small-cell lung cancer. BMJ Open Respir Res. (2023) 10:e001492. doi: 10.1136/bmjresp-2022-001492 PMC10277533 37321664

[3] Yang Y Wang Y. Targeting exon 20 insertion mutations in lung cancer. Curr Opin Oncol. (2023) 35:37–45. doi: 10.1097/CCO.0000000000000919 36380577

[4] Zhou C Ramalingam SS Kim TM Kim SW Yang JC Riely GJ. Treatment outcomes and safety of mobocertinib in platinum-pretreated patients with EGFR exon 20 insertion-positive metastatic non-small cell lung cancer: A phase 1/2 open-label nonrandomized clinical trial. JAMA Oncol. (2021) 7:e214761. doi: 10.1001/jamaoncol.2021.4761 34647988 PMC8517885

[5] Chon K Larkins E Chatterjee S Mishra-Kalyani PS Aungst S Wearne E. FDA approval summary: amivantamab for the treatment of patients with non-small cell lung cancer with EGFR exon 20 insertion mutations. Clin Cancer Res. (2023) 29:3262–6. doi: 10.1158/1078-0432.CCR-22-3713 PMC10523842 37022784

[6] Brazel D Kroening G Nagasaka M. Non-small cell lung cancer with EGFR or HER2 exon 20 insertion mutations: diagnosis and treatment options. BioDrugs. (2022) 36:717–29. doi: 10.1007/s40259-022-00556-4 PMC9649507 36255589

[7] Shi Y Chen G Wang X Liu Y Wu L Hao Y. Furmonertinib (AST2818) versus gefitinib as first-line therapy for Chinese patients with locally advanced or metastatic EGFR mutation-positive non-small-cell lung cancer (FURLONG): a multicentre, double-blind, randomised phase 3 study. Lancet Respir Med. (2022) 10:1019–28. doi: 10.1016/S2213-2600(22)00168-0 35662408

[8] Han B Zhou C Wu L Yu X Li Q Liu F. 1210P Preclinica l and preliminary clinical investigations of furmonertinib in NSCLC with EGFR exon 20 insertions (20ins). Ann Oncol. (2021) 32:s964. doi: 10.1016/j.annonc.2021.08.1815

[9] Yasuda H Park E Yun CH Sng NJ Lucena-Araujo AR Yeo WL. Structural, biochemical, and clinical characterization of epidermal growth factor receptor (EGFR) exon 20 insertion mutations in lung cancer. Sci Transl Med. (2013) 5:216ra177. doi: 10.1126/scitranslmed.3007205 PMC3954775 24353160

[10] Riess JW Gandara DR Frampton GM Madison R Peled N Bufill JA. Diverse EGFR exon 20 insertions and co-occurring molecular alterations identified by comprehensive genomic profiling of NSCLC. J Thorac Oncol. (2018) 13:1560–8. doi: 10.1016/j.jtho.2018.06.019 PMC6764748 29981927

[11] Yang G Li J Xu H Yang Y Yang L Xu F. EGFR exon 20 insertion mutations in Chinese advanced non-small cell lung cancer patients: Molecular heterogeneity and treatment outcome from nationwide real-world study. Lung Cancer. (2020) 145:186–94. doi: 10.1016/j.lungcan.2020.03.014 32336530

[12] Jiao XD Qin BD You P Cai J Zang YS. The prognostic value of TP53 and its correlation with EGFR mutation in advanced non-small cell lung cancer, an analysis based on cBioPortal data base. Lung Cancer. (2018) 123:70–5. doi: 10.1016/j.lungcan.2018.07.003 30089598

[13] Fu Y Wang A Zhou J Feng W Shi M Xu X. Advanced NSCLC patients with EGFR T790M harboring TP53 R273C or KRAS G12V cannot benefit from osimertinib based on a clinical multicentre study by tissue and liquid biopsy. Front Oncol. (2021) 11:621992. doi: 10.3389/fonc.2021.621992 33718183 PMC7943858

[14] Vokes NI Chambers E Nguyen T Coolidge A Lydon CA Le X. Concurrent TP53 mutations facilitate resistance evolution in EGFR-mutant lung adenocarcinoma. J Thorac Oncol. (2022) 17:779–92. doi: 10.1016/j.jtho.2022.02.011 PMC10478031 35331964

[15] Chen D Song Z Cheng G. Clinical efficacy of first-generation EGFR-TKIs in patients with advanced non-small-cell lung cancer harboring EGFR exon 20 mutations. Onco Targets Ther. (2016) 9:4181–6. doi: 10.2147/OTT.S108242 PMC4944908 27468240

[16] Fang W Huang Y Hong S Zhang Z Wang M Gan J. EGFR exon 20 insertion mutations and response to osimertinib in non-small-cell lung cancer. BMC Cancer. (2019) 19:595. doi: 10.1186/s12885-019-5820-0 31208370 PMC6580637

[17] Piotrowska Z Wang Y Sequist LV Ramalingam SS. ECOG-ACR IN 5162: A phase II study of osimertinib 160 mg in NSCLC with EGFR exon 20 insertions. J Clin Oncol. (2020) 38:9513. doi: 10.1200/jco.2020.38.15_suppl.9513

[18] Van Veggel B Madeira R Santos J Hashemi S Paats MS Monkhorst K Heideman D. Osimertinib treatment for patients with EGFR exon 20 mutation positive non-small cell lung cancer. Lung Cancer. (2020) 141:9–13. doi: 10.1016/j.lungcan.2019.12.013 31926441

[19] Riely GJ Neal JW Camidge DR Spira AI Piotrowska Z Costa DB. Activity and safety of mobocertinib (TAK-788) in previously treated non-small cell lung cancer with EGFR exon 20 insertion mutations from a phase I/II trial. Cancer Discovery. (2021) 11:1688–99. doi: 10.1158/2159-8290.CD-20-1598 PMC8295177 33632775

[20] Ramalingam SS Zhou C K Kim TM Yang JC Riely GJ Mekhail T. 988P Phase I/II study of mobocertinib in EGFR exon 20 insertion (ex20ins) + metastatic NSCLC (mNSCLC): Updated results from platinum-pretreated patients (PPP). Ann Oncol. (2022) 33:S1004. doi: 10.1016/j.annonc.2022.07.1115

[21] Han B Zhou C Zheng W Wu L Ma Z Wang H. A phase 1b study of furmonertinib, an oral, brain penetrant, selective EGFR inhibitor, in patients with advanced NSCLC with EGFR exon 20 insertions, in: Presented at: International Association for the Study of Lung Cancer 2023 World Conference on Lung Cancer, *J Thorac Oncol* . (2023) 18: S49. doi: 10.1016/j.jtho.2023.09.033

[22] Zhou X Dong H Li P Wu C Lu H Xiao M. Short-term efficacy of furmonertinib in treatment of NSCLC patients with EGFR exon20 insertion. J Clin Oncol. (2022) 40:e21063. doi: 10.1200/JCO.2022.40.16_suppl.e21063

[23] Elamin YY Robichaux JP Carter BW Altan M Tran H Gibbons DL. Poziotinib for EGFR exon 20-mutant NSCLC: Clinical efficacy, resistance mechanisms, and impact of insertion location on drug sensitivity. Cancer Cell. (2022) 40:754–67.e6. doi: 10.1016/j.ccell.2022.06.006 35820397 PMC9667883

